# Integrating clinical, laboratory and quantitative CT features for predicting split renal function in urinary tract obstruction

**DOI:** 10.1186/s12880-026-02352-w

**Published:** 2026-04-16

**Authors:** Ying Ma, Jie Zhan, Jiajun Feng, Wanli Zhang, Fangrong Liang, Honggang Xu, Yixiu Hao, Long Qu, Wenjuan He, Shengsheng Lai, Ruimeng Yang

**Affiliations:** 1 Department of Radiology, Guangzhou First People’s Hospital, School of Medicine, South China University of Technology, Guangzhou, Guangdong 510180 China; 2 Department of Radiology, The Second Affiliated Hospital, School of Medicine, South China University of Technology, Guangzhou, Guangdong 510180 China; 3 School of Medicine, South China University of Technology, Guangzhou, Guangdong 510006 China; 4 School of Medical Equipment, Guangdong Food and Drug Vocational College, Guangzhou, Guangdong 510520 China

**Keywords:** Split renal function, Urinary tract obstruction, Contrast-enhanced computed tomography, Renal cortical thickness

## Abstract

**Background:**

Urinary tract obstruction (UTO) can lead to progressive renal impairment, making accurate evaluation of split renal function (SRF) essential for clinical decision-making. Although radionuclide renal dynamic scintigraphy remains the gold standard for SRF assessment, its clinical application is constrained by procedural complexity, limited availability, and sensitivity to anatomical variations. Thus, there is a clinical need for simpler, reliable, and noninvasive alternative approaches. This study aimed to develop and validate a predictive model for SRF grading by integrating clinical variables, laboratory parameters, and quantitative contrast-enhanced computed tomography (CECT) features in patients with UTO.

**Methods:**

A retrospective cohort of 78 patients with UTO (150 kidneys) was analyzed. Based on split renal glomerular filtration rate (GFR) determined using the Gates method, kidneys were categorized into normal, mild-to-moderate impairment, and severe impairment groups. Clinical variables, laboratory parameters, and quantitative CECT features were collected. Univariate and multivariate logistic regression analyses were performed to identify independent predictors and construct SRF grading models. Model performance was evaluated using the area under the receiver operating characteristic curve (AUC) and decision curve analysis (DCA).

**Results:**

In Task 1 (normal vs. abnormal) and Task 2 (normal vs. mild-to-moderate), age was the key univariate predictor, while hemoglobin (Hb) and renal cortical thickness (Rc) were identified as independent predictors in the laboratory-based and CECT-based multivariate models, respectively. For Task 1, the Combined model [Clinical predictor (Age) + Laboratory model (Hb-based) + CECT model (Rc-based)] achieved the highest diagnostic performance (AUC = 0.890); for Task 2, the optimal model was [Clinical predictor (Age) + CECT model (Rc-based)] (AUC = 0.810). For Task 3 (mild-to-moderate vs. severe), Hb was the strongest univariate predictor, and Rc was the sole independent predictor in the CECT-based multivariate model. The highest diagnostic accuracy for Task 3 was achieved by the combined Laboratory predictor (Hb) + CECT model (Rc-based), with an AUC of 0.970.

**Conclusion:**

The CECT model (Rc-based) serves as a crucial imaging biomarker for evaluating SRF impairment in patients with UTO. Task-specific models combining the CECT model (Rc-based) with a clinical predictor (Age) and laboratory information—either as a univariable predictor (Hb) or as a multivariable laboratory model (Hb-based)—showed superior predictive performance. This integrated, noninvasive strategy may serve as a useful adjunct to radionuclide imaging for individualized SRF assessment, pending prospective validation.

**Supplementary Information:**

The online version contains supplementary material available at 10.1186/s12880-026-02352-w.

## Introduction

Urinary tract obstruction (UTO) is a major cause of progressive renal impairment [1]. Following obstruction, inflammatory infiltration and interstitial fibrosis may lead to irreversible renal parenchymal injury and nephron loss, ultimately resulting in functional decline [2]. In this context, accurate assessment of split renal function (SRF) is essential for determining the optimal timing of intervention, evaluating contralateral compensatory capacity, and guiding individualized treatment strategies.

Currently, renal dynamic scintigraphy using ^99m^Tc-diethylenetriaminepentaacetic acid (^99m^Tc-DTPA), combined with the Gates method for calculating split renal glomerular filtration rate (GFR), is widely regarded as the clinical reference standard for SRF assessment [3]. However, this technique is technically demanding, not universally available, and its accuracy can be affected by renal position, morphology, and region-of-interest delineation, particularly in patients with anatomical abnormalities. Notably, some patients with upper UTO preoperatively classified as having severe impairment may exhibit partial recovery of GFR after relief of obstruction [4], suggesting that scintigraphy may underestimate residual renal function and potentially lead to overly aggressive treatment.

Contrast-enhanced computed tomography (CECT) is routinely performed in patients with UTO for etiological evaluation. Beyond identifying the cause and extent of obstruction, CECT provides high-resolution anatomical information and allows indirect assessment of renal perfusion and excretory function [5]. Previous studies reported a strong correlation between CECT-derived GFR and scintigraphy-based GFR, suggesting that CECT can simultaneously evaluate urinary tract morphology and SRF [6]. In addition, quantitative renal morphological parameters obtained from CECT—such as cortical thickness, parenchymal thickness, and parenchymal volume—have been shown to correlate with renal function across various clinical settings [7–10].

Nevertheless, most existing studies have focused on simple correlations between individual imaging parameters and renal function [9, 10], without developing integrated predictive models that combine quantitative CECT features with clinical and laboratory variables. This limitation is particularly relevant in patients with UTO, in whom SRF assessment should not only characterize the degree of functional impairment but also support clinical decision-making regarding renal preservation or intervention.

Therefore, this study aimed to develop and validate predictive models for SRF grading in patients with UTO by integrating quantitative morphological features extracted from routine CECT images with key clinical and laboratory parameters. By establishing a simple, reliable, and noninvasive assessment framework, this study may offer a useful adjunct to radionuclide imaging, particularly in clinical settings with limited access to nuclear medicine facilities.

## Materials and methods

### Patients

This retrospective study was approved by the Medical Ethics Committee of Guangzhou First People’s Hospital, School of Medicine, South China University of Technology (Approval No. K-2022-076-04) and conducted in accordance with the Declaration of Helsinki. The study followed the Strengthening the Reporting of Observational Studies in Epidemiology (STROBE) guidelines (Supplementary Table 1).

Between January 2017 and August 2020, medical records of 111 consecutive patients with a clinical diagnosis of UTO were retrospectively reviewed. Inclusion criteria were: (1) complete clinical and laboratory data; (2) availability of contrast-enhanced abdominal CT with satisfactory image quality, including unenhanced, corticomedullary, and excretory phases; and (3) completion of ⁹⁹ᵐTc-DTPA dynamic renal scintigraphy. Exclusion criteria included: (1) conditions known to significantly affect renal function assessment, such as diabetic nephropathy, uncontrolled hypertension (persistent blood pressure > 140/90 mmHg), hematologic disorders, or hemoglobin abnormalities; (2) renal diseases interfering with imaging analysis or functional evaluation (e.g., polycystic kidney disease, renal abscess, or renal tumors); and (3) the presence of a nephrostomy tube or ureteral stent at the time of imaging. Patients with diabetes mellitus or hypertension without evidence of diabetic nephropathy or uncontrolled blood pressure were eligible for inclusion.

After screening, 33 patients were excluded (32 with coexisting renal parenchymal lesions and 1 with a ureteral stent), leaving 78 eligible patients for final analysis. Six patients had a solitary kidney due to prior nephrectomy, resulting in a total of 150 kidneys available for analysis. The etiologies of obstruction included renal calculi (*n* = 49), inflammatory ureteric strictures (*n* = 45), and tumor-related ureteral strictures (*n* = 6). Unobstructed contralateral kidneys were included for comparative analysis and served as reference kidneys, with functional status determined according to scintigraphic criteria.

**Fig. 1:**
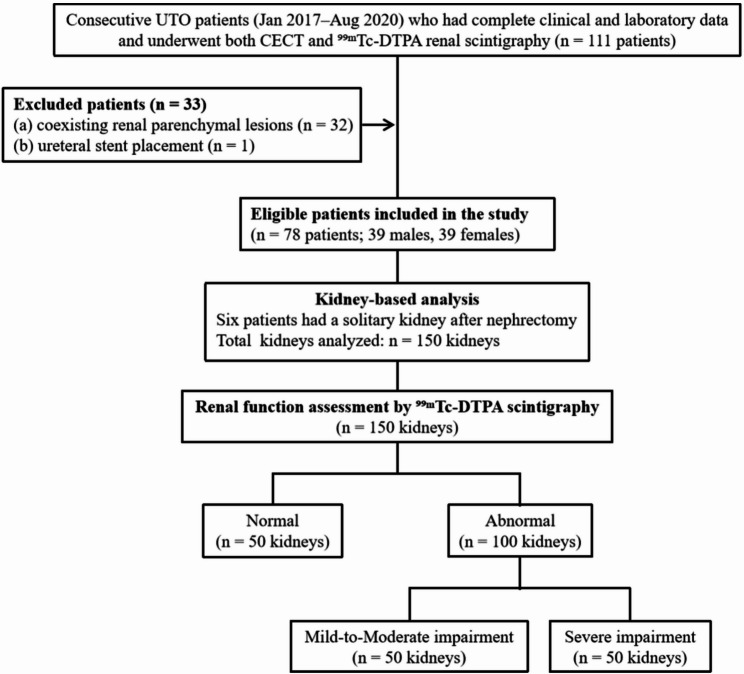
Flowchart of patient selection

All patients underwent CECT within one week of radionuclide renal scintigraphy. For patients with baseline renal insufficiency (estimated GFR < 60 mL/min/1.73 m²), prophylactic hydration was administered prior to CT examination in accordance with institutional protocols. The patient selection process is summarized in Fig. 1.

### Clinical data collection

Clinical and laboratory data were retrieved from the hospital’s electronic medical record system. Collected variables included sex, laterality (left/right), age, body weight, white blood cell (WBC) count, hemoglobin (Hb), serum creatinine (Scr), blood urea nitrogen (BUN), and estimated glomerular filtration rate (eGFR). All laboratory tests were performed within one week of CECT and scintigraphic examinations.

### Imaging acquisition and analysis

#### ^99 m^ Tc-DTPA renal dynamic scintigraphy

All scintigraphic examinations were performed using a GE Infinia Hawkeye 4 SPECT/CT system (GE Healthcare, USA). Patients were instructed to empty their bladder prior to imaging. After intravenous administration of ^99m^Tc-DTPA (185–370 MBq), dynamic images were acquired over a 20-minute period to assess renal perfusion and function. Time–activity curves were generated using the manufacturer’s software, and total and split renal GFR values were automatically calculated using the Gates method.

According to the age-specific lower limit of normal (LLN) for total GFR provided in the GE operator manual, kidneys were initially categorized as having either normal or abnormal function. The abnormal group was further stratified into mild, moderate, and severe impairment based on the following criteria (refer to Table 1): Normal, total GFR ≥ LLN; Mild impairment, < LLN but ≥ 2/3 LLN; Moderate impairment, < 2/3 LLN but ≥ 1/3 LLN; and Severe impairment, < 1/3 LLN. The reference value for SRF was defined as one-half of the age-specific total GFR reference value. This grading scheme reflects physiological age-related variation in renal function and provides a standardized framework for SRF stratification.

**Table 1:** Reference ranges for GFR measured by renal scintigraphy in healthy adults

Age (year)	Mean value (mL/min)	Lower normal limit (mL/min)
20–29	115	88
30–39	106	86
40–49	104	80
50–59	99	75
> 59	96	73

Renal function grading: Normal (≥ age-specific lower limit of total GFR); Mild impairment (< age-specific lower limit but ≥ 2/3 of the limit); Moderate impairment (< 2/3 of the limit but ≥ 1/3 of the limit); Severe impairment (< 1/3 of the limit). Split renal GFR reference values were derived as 50% of the total GFR values; GFR, glomerular filtration rate

#### Abdominal contrast-enhanced CT (CECT)

CECT examinations were performed using three multidetector CT scanners (Toshiba 16-slice, Toshiba 320-slice, and Philips 64-slice scanners). Standardized parameters were applied: 120 kVp, 200 mAs, and 5-mm slice thickness. A nonionic iodinated contrast agent (Iopromide, 370 mg I/mL) was administered intravenously at a dose of 2.0 mL/kg and an injection rate of 3–4 mL/s via the antecubital vein. CT images were acquired in unenhanced, corticomedullary (30–40 s post-injection), and excretory (5–10 min post-injection) phases. All image datasets were exported in Digital Imaging and Communications in Medicine (DICOM) format, anonymized, and archived for subsequent quantitative analysis.

### Quantitative CECT feature extraction

#### Two-dimensional morphometric analysis

**Fig. 2:**
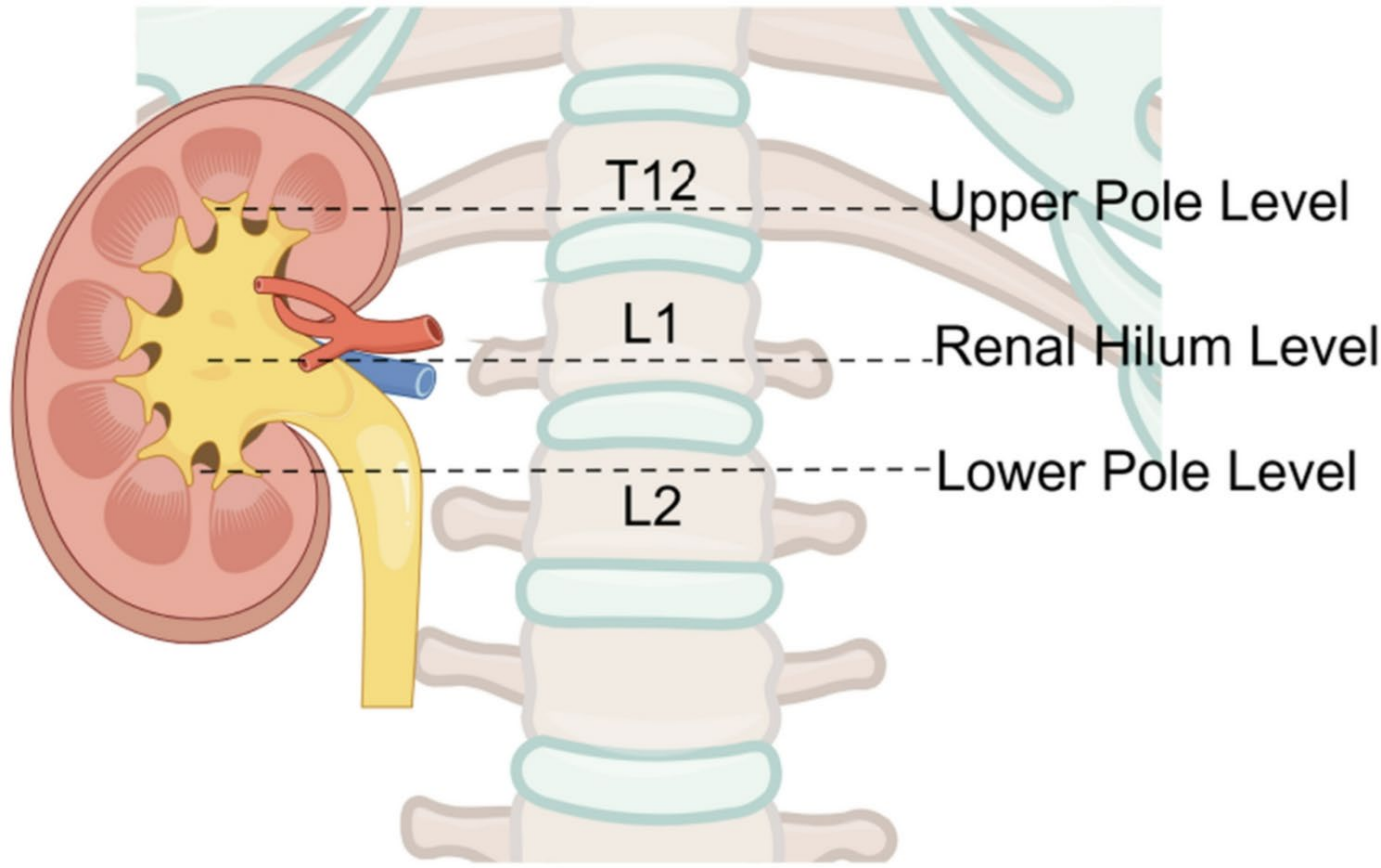
Schematic diagram of the measurement levels. The upper pole, middle section, and lower pole of the kidney are defined as the levels of the upper renal calyces, renal hilum, and lower renal calyces, respectively

**Fig. 3:**
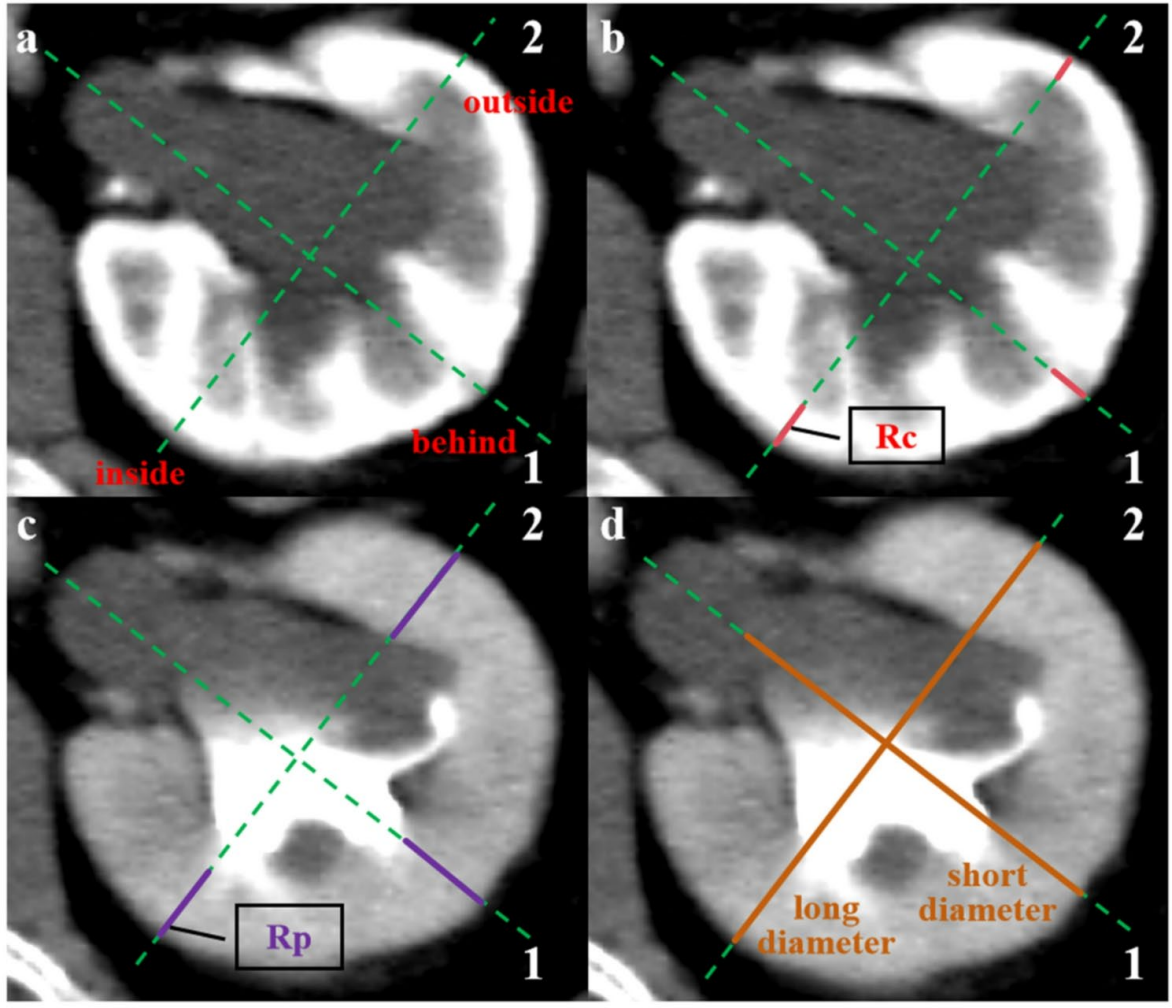
Methodology for quantifying renal features on contrast-enhanced CT (CECT). **(a)** Schematic showing the renal axis at the hilar level; **(b)** Measurement of renal cortical thickness (red line) on a corticomedullary phase image, defined as the shortest distance between the inner and outer cortical edges; **(c)** Measurement of renal parenchymal thickness (purple line) on an excretory phase image; **(d)** Determination of the kidney size index (orange lines) at the hilar level, calculated as the sum of the long and short diameters

Two board-certified radiologists (Y Ma and RM Yang, with 6 and 15 years of experience, respectively) independently analyzed all CT images while blinded to clinical and scintigraphic findings. Each parameter was measured three times, and the mean value was used for analysis. Any discrepancies were resolved by consensus with a senior radiologist. Measurements were performed using RadiAnt DICOM Viewer (https://www.radiantviewer.com/). Three standardized axial levels were selected for analysis: the upper calyceal level, the renal hilum, and the lower calyceal level (Fig. 2). At the renal hilum, two perpendicular axes intersecting at the midpoint of the renal pelvis were drawn and used as reference lines for measurements at other levels (Fig. 3a).

The following morphometric indices were obtained:

- Renal cortical thickness (Rc) and renal parenchymal thickness (Rp): measured as the shortest cortical and parenchymal diameters along each axis on corticomedullary-phase and excretory-phase images, respectively (Fig. 3b and c). Nine measurements were obtained per kidney, and the mean value for each parameter was calculated.
- Renal parenchymal thickness index (Rp-I): defined as the sum of parenchymal thickness measurements along both axes at the renal hilum on excretory-phase images (Fig. 3c).
- Renal size index (R-I): defined as the sum of the long and short renal diameters measured at the renal hilum (Fig. 3d).

#### Three-dimensional volumetric analysis

**Fig. 4:**
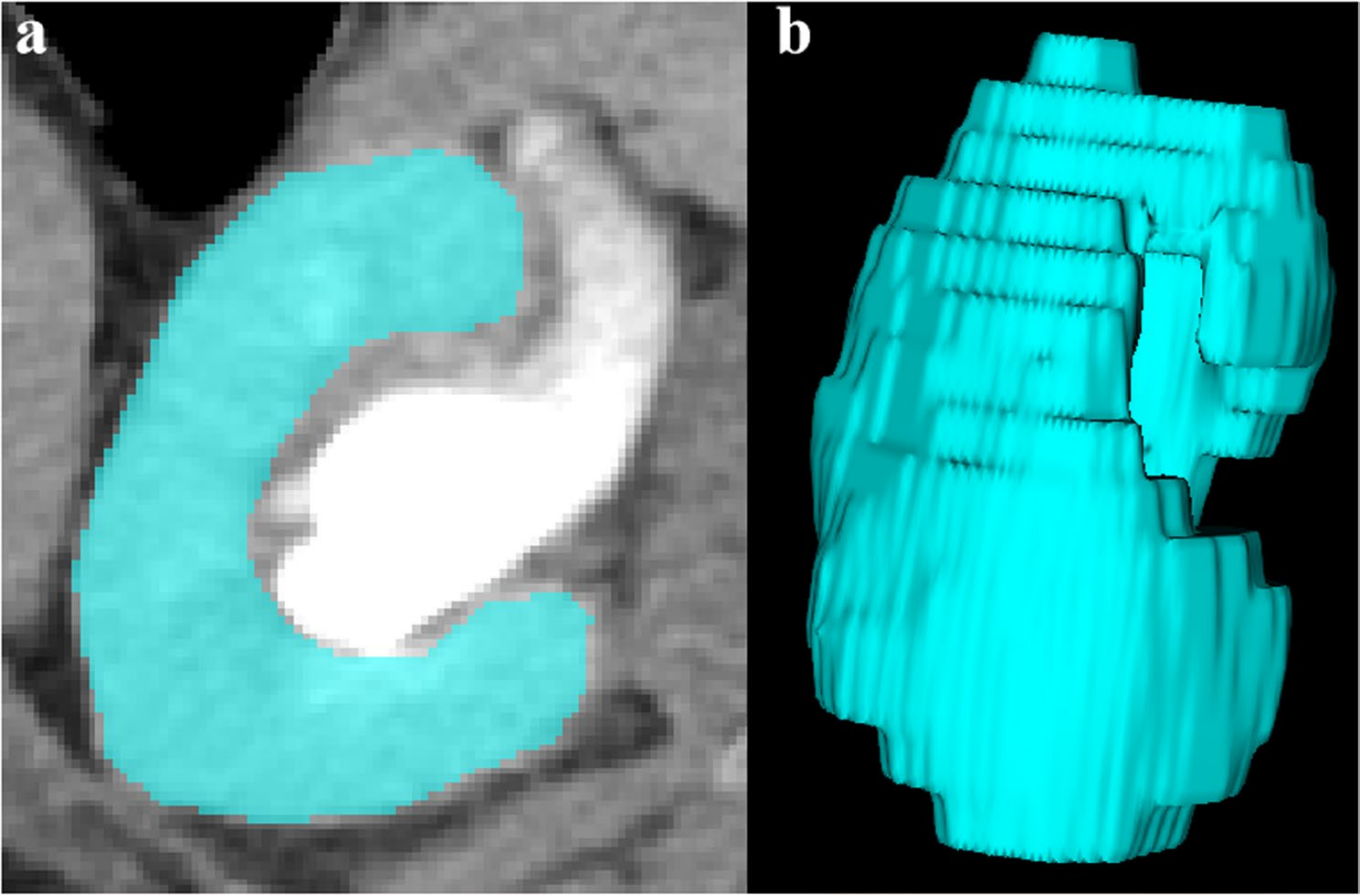
Renal volume of interest (VOI) on excretory phase CT. **(a)** Axial image at the renal hilum; **(b)** 3D representation of the segmented VOI

Renal parenchymal volume (RPV) was quantified using ITK-SNAP software (http://www.itksnap.org). The excretory phase was selected to optimize contrast between the enhanced renal parenchyma and the opacified collecting system. The volume of interest (VOI) encompassing the entire renal parenchyma was manually segmented slice by slice, with careful exclusion of renal sinus fat, hilar vessels, pelvis, calyces, cysts, and calculi (Fig. 4). The final renal parenchymal volume was automatically calculated by the software.

#### Interobserver reproducibility

Interobserver reproducibility of all manual morphological measurements was assessed using the intraclass correlation coefficient (ICC) for two-dimensional parameters and the Dice similarity coefficient (DSC) for three-dimensional renal parenchymal volume segmentation. Agreement thresholds were predefined as ICC > 0.75 and DSC > 0.90. Any cases not meeting these thresholds were jointly reviewed and resolved by consensus.

### Statistical analysis

All statistical analyses were performed using SPSS software (version 25.0; IBM Corp.). Continuous variables were assessed for normality using the Kolmogorov-Smirnov test (*n* > 50) or the Shapiro-Wilk test (*n* ≤ 50). Normally distributed data are presented as mean ± standard deviation and compared using one-way analysis of variance (ANOVA), whereas non-normally distributed data are expressed as median (interquartile range) and compared using the Kruskal-Wallis *H* test. Categorical variables are reported as number (proportion) and compared using the chi-square test. Bonferroni correction was applied for multiple comparisons, and a two-tailed *p* < 0.05 was considered statistically significant.

To stratify renal function, multivariate logistic regression models were constructed for three predictive tasks: (1) discriminating normal from abnormal kidneys, (2) distinguishing normal from mild-to-moderate impairment, and (3) differentiating mild-to-moderate from severe impairment. Variables were categorized into three groups: clinical factors (sex, laterality, age, weight), laboratory parameters (WBC, Hb, Scr, BUN, eGFR), and CECT features (Rc, Rp, Rp-I, R-I, RPV). Variables with significant intergroup differences (*p <* 0.05) in univariate analysis were initially included. Multicollinearity was assessed using the variance inflation factor (VIF), and variables with VIF < 5 were retained. Based on univariate and multivariate logistic regression analyses, a series of single and combined prediction models with different variable combinations were developed to determine the optimal model for each research objective. Model performance was evaluated using receiver operating characteristic (ROC) curve analysis, with the area under the ROC curve (AUC) used to assess discriminative ability. Differences in AUC were compared using DeLong’s test, with a two-tailed *p* < 0.05 considered statistically significant. To evaluate the stability and generalizability of the predictive models for each classification task, internal validation was performed using 1,000 bootstrap resamples. The clinical utility of the models was further assessed via decision curve analysis (DCA).

## Results

### Patient characteristics

A total of 150 kidneys from 78 patients with UTO (39 males and 39 females; median age, 59.0 years) were included. Based on SRF assessed by ⁹⁹ᵐTc-DTPA scintigraphy, kidneys were categorized into three groups: normal, mild-to-moderate impairment, and severe impairment, with 50 kidneys in each group.

No significant intergroup differences were observed in sex, body weight, obstruction etiology, or laterality (all *p* > 0.05). In contrast, age and laboratory parameters—including Hb, Scr, BUN, and eGFR—differed significantly among the three renal function groups (all *p* < 0.05). Detailed clinical and laboratory characteristics are summarized in Table 2.

**Table 2:** Comparison of clinical, laboratory, and CECT features across renal function groups

Variables		Abnormal		
	Normal (*n* = 50)	Mild-to-Moderate (*n* = 50)	Severe (*n* = 50)	*p*
**Clinical variables**				
Sex, n (%)				0.487
Male	25 (50)	28 (56)	22 (44)	
Female	25 (50)	22 (44)	28 (56)	
Laterality, n (%)				0.513
Left	24 (48)	24 (48)	29 (58)	
Right	26 (52)	26 (52)	21 (42)	
Age (year)	56.0 (47.8, 64.8)	62.0 ± 11.3	61.5 (52.0, 70.3)	**0.038**
Weight (kg)	56.1 ± 9.8	58.5 ± 9.9	56.6 ± 10.2	0.440
**Laboratory parameters**				
WBC (10^9^/L)	6.6 (5.6, 8.1)	6.4 (4.9, 8.3)	6.7 (5.9, 8.8)	0.542
Hb (g/L)	133.0 (121.8, 144.0)	121.8 ± 19.5	124.0 ± 24.4	**0.041**
Scr (μmol/L)	87.5 (66.8, 101.3)	96.5 (73.0, 138.8)	99.5 (81.0, 141.5)	**0.011**
BUN (mmol/L)	5.2 (4.4, 6.7)	6.5 (4.8, 8.0)	6.6 (4.9, 8.1)	**0.024**
eGFR (mL/min/1.73m^2^)	78.1 (60.4, 93.6)	65.5 ± 26.8	63.1 ± 23.8	**0.004**
**CECT features**				
Rc (mm)	7.0 (6.0, 8.0)	5.4 ± 2.2	2.0 (2.0, 3.0)	**< 0.001**
Rp (mm)	21.0 (18.0, 23.0)	18.0 (11.0, 18.0)	5.0 (3.0, 10.0)	**< 0.001**
Rp-I (mm)	62.5 (54.0, 68.5)	54.5 (31.5, 63.3)	15.5 (8.9, 30.0)	**< 0.001**
R-I (mm)	114.9 ± 16.3	111.5 (100.8, 123.3)	120.5 (92.5, 166.0)	0.466
RPV (mm^3^)	175750.0 ± 37616.0	135974.5 (104867.0, 167407.0)	61076.0 (37153.0, 88164.0)	**< 0.001**
**Cause of urinary tract obstruction**,** n (%)**				0.661
Renal calculi	0 (0)	25 (50)	24 (48)	
Inflammatory ureteric strictures	0 (0)	21 (42)	24 (48)	
Tumor-related ureteral strictures	0 (0)	4 (8)	2 (4)	

Continuous variables are presented as mean (± standard deviation, SD) or median (interquartile range, IQR) and were compared using one-way analysis of variance (ANOVA) or the Kruskal-Wallis *H* test; categorical variables are presented as number (proportion) and were compared using the chi-squared test; WBC, white blood cell; Hb, hemoglobin; Scr, serum creatinine; BUN, blood urea nitrogen; eGFR, estimated glomerular filtration rate; Rc, renal cortical thickness; Rp, renal parenchymal thickness; Rp-I, renal parenchymal thickness index; R-I, renal size index; RPV, renal parenchymal volume; *p* < 0.05 is indicated by bold type

### Analysis of quantitative CECT features

Comparisons of quantitative CECT-derived parameters among renal function groups are summarized in Table 2. Rc, Rp, Rp-I, and RPV demonstrated significant differences across the three SRF groups (all *p* < 0.001), and exhibited progressive reductions with worsening renal function. In contrast, R-I did not differ significantly among the groups (*p* = 0.466).

### Development and performance of SRF prediction models

To develop SRF prediction models, multivariate logistic regression analyses were performed using laboratory parameters and CECT features that demonstrated significant associations with SRF in univariate analyses (Tables 3, 4 and 5). Due to strong collinearity between Rp and Rp-I (refer to Supplementary Table 2), only Rp—the more representative variable—was retained for model development.

**Table 3:** Univariate and multivariate logistic regression analyses for differentiating normal from abnormal renal function

Variables	Univariate analysis			Model 1 (Multivariate analysis-Laboratory parameters)			Model 2 (Multivariate analysis-CECT features)		
	β coefficient	OR (95% CI)	*p*	β coefficient	OR (95% CI)	*p*	β coefficient	OR (95% CI)	*p*
**Clinical variables**									
Age (year)	0.032	1.033 (1.009, 1.058)	**0.008**						
**Laboratory parameters**									
Hb (g/L)	-0.019	0.981 (0.965, 0.998)	**0.028**	-0.019	0.982 (0.964, 0.999)	**0.047**			
Scr (μmol/L)	0.015	1.015 (1.005, 1.026)	**0.005**	0.004	1.004 (0.985, 1.023)	0.713			
BUN (mmol/L)	0.223	1.250 (1.053, 1.484)	**0.011**	0.101	1.107 (0.865, 1.416)	0.420			
eGFR (mL/min/1.73m^2^)	-0.025	0.975 (0.962, 0.989)	**< 0.001**	-0.015	0.985 (0.962, 1.009)	0.228			
**CECT features**									
Rc (mm)	-0.758	0.469 (0.364, 0.604)	**< 0.001**				-0.513	0.599 (0.413, 0.869)	**0.007**
Rp (mm)	-0.237	0.789 (0.724, 0.860)	**< 0.001**				-0.095	0.909 (0.800, 1.034)	0.146
RPV (mm^3^)	-2.117 × 10^− 5^	99.998 × 10^− 2^ (99.997 × 10^− 2^, 99.999 × 10^− 2^)	**< 0.001**				^−2.154 × 10^ *-6*	999.998 × 10^− 3^ (99.999 × 10^− 2^, 10.000 × 10^− 1^)	0.736

Hb, hemoglobin; Scr, serum creatinine; BUN, blood urea nitrogen; eGFR, estimated glomerular filtration rate; Rc, renal cortical thickness; Rp, renal parenchymal thickness; RPV, renal parenchymal volume; OR, odds ratio; CI, confidence interval; *p* < 0.05 is indicated by bold type

**Table 4:** Univariate and multivariate logistic regression analyses for differentiating normal from mild-to-moderate renal function impairment

**Variables**	**Univariate analysis**			**Model 1 (Multivariate analysis-Laboratory parameters)**			**Model 2 (Multivariate analysis-CECT features)**		
	β coefficient	**OR (95% CI)**	***p***	β coefficient	**OR (95% CI)**	***p***	β coefficient	**OR (95% CI)**	***p***
**Clinical variables**									
Age (year)	0.044	1.045 (1.012, 1.079)	**0.007**						
**Laboratory parameters**									
Hb (g/L)	-0.024	0.976 (0.956, 0.997)	**0.025**	-0.023	0.977 (0.956, 0.999)	**0.039**			
Scr (μmol/L)	0.014	1.014 (1.002, 1.025)	**0.019**	0.004	1.004 (0.982, 1.026)	0.742			
BUN (mmol/L)	0.209	1.233 (1.002, 1.511)	**0.044**	0.105	1.111 (0.826, 1.493)	0.487			
eGFR (mL/min/1.73m^2^)	-0.022	0.978 (0.962, 0.994)	**0.007**	-0.012	0.989 (0.961, 1.016)	0.416			
**CECT features**									
Rc (mm)	-0.543	0.581 (0.439, 0.770)	**< 0.001**				-0.387	0.679 (0.465, 0.992)	**0.045**
Rp (mm)	-0.167	0.847 (0.770, 0.931)	**< 0.001**				-0.080	0.923 (0.808, 1.054)	0.237
RPV (mm^3^)	-1.527 × 10 **− 5**	99.998 × 10^− 2^ (99.998 × 10^− 2^, 99.999 × 10^− 2^)	**0.002**				-0.481 × 10^− 6^	9999.995 × 10^− 4^ (999.986 × 10^− 3^, 1000.013 × 10^− 3^)	0.945

Hb, hemoglobin; Scr, serum creatinine; BUN, blood urea nitrogen; eGFR, estimated glomerular filtration rate; Rc, renal cortical thickness; Rp, renal parenchymal thickness; RPV, renal parenchymal volume; OR, odds ratio; CI, confidence interval; *p* < 0.05 is indicated by bold type

**Table 5:** Univariate and multivariate logistic regression analyses for differentiating mild-to-moderate from severe renal function impairment

Variables	Univariate analysis			Model 2 (Multivariate analysis-CECT features)		
	β coefficient	OR (95% CI)	*p*	β coefficient	OR (95% CI)	*p*
**Clinical variables**						
Age (year)	-0.014	0.986 (0.957, 1.016)	0.346			
**Laboratory parameters**						
Hb (g/L)	0.089	1.093 (1.055, 1.132)	**< 0.001**			
Scr (μmol/L)	0.002	1.002 (0.996, 1.008)	0.528			
BUN (mmol/L)	0.029	1.030 (0.938, 1.131)	0.539			
eGFR (mL/min/1.73m^2^)	-0.004	0.996 (0.981, 1.012)	0.634			
**CECT features**						
Rc (mm)	-1.105	0.331 (0.215, 0.510)	**< 0.001**	-0.814	0.443 (0.241, 0.814)	**0.009**
Rp (mm)	-0.232	0.793 (0.728, 0.864)	**< 0.001**	-0.045	0.956 (0.837, 1.092)	0.504
RPV (mm^3^)	-2.447 × 10^− 5^	99.998 × 10^− 2^ (99.997 × 10^− 2^, 99.999 × 10^− 2^)	**< 0.001**	^−1.042^ × ^10−5^	99.999 × 10^− 2^ (99.998 × 10^− 2^, 10.000 × 10^− 1^)	0.104

Hb, hemoglobin; Scr, serum creatinine; BUN, blood urea nitrogen; eGFR, estimated glomerular filtration rate; Rc, renal cortical thickness; Rp, renal parenchymal thickness; RPV, renal parenchymal volume; OR, odds ratio; CI, confidence interval; *p* < 0.05 is indicated by bold type

Three prediction tasks were established to evaluate model performance for SRF grading:

#### Task 1: Normal vs. abnormal renal function

Univariate and multivariate analyses revealed that age, Hb, Scr, BUN, eGFR, Rc, Rp, and RPV were all significantly associated with SRF (all *p* < 0.05). Hb was an independent predictor in the laboratory parameters model (OR = 0.982, 95% CI: 0.964–0.999, *p* = 0.047). In the CECT features model, Rc was the strongest independent predictor (OR = 0.599, 95% CI: 0.413–0.869, *p* = 0.007), while neither Rp nor RPV demonstrated independent significance.

#### Task 2: Normal vs. mild-to-moderate renal impairment

Univariate and multivariate analyses revealed that age, Hb, Scr, BUN, eGFR, Rc, Rp, and RPV were significantly associated with SRF (all *p <* 0.05). In the laboratory parameters model, Hb was the sole variable with independent predictive value (OR = 0.977, 95% CI: 0.956–0.999, *p* = 0.039). In the CECT features model, Rc again demonstrated strong independent predictive value (OR = 0.679, 95% CI: 0.465–0.992, *p* = 0.045), whereas neither Rp nor RPV showed independent significance.

#### Task 3: Mild-to-moderate vs. severe renal impairment

Univariate and multivariate analyses revealed that Hb, Rc, Rp, and RPV were significantly associated with SRF (all *p <* 0.05). The CECT features model revealed that Rc remained an independent predictor (OR = 0.443, 95% CI: 0.241–0.814, *p* = 0.009), and could effectively discriminate between mild-to-moderate and severe renal impairment.

### Comparison of model performance

**Fig. 5:**
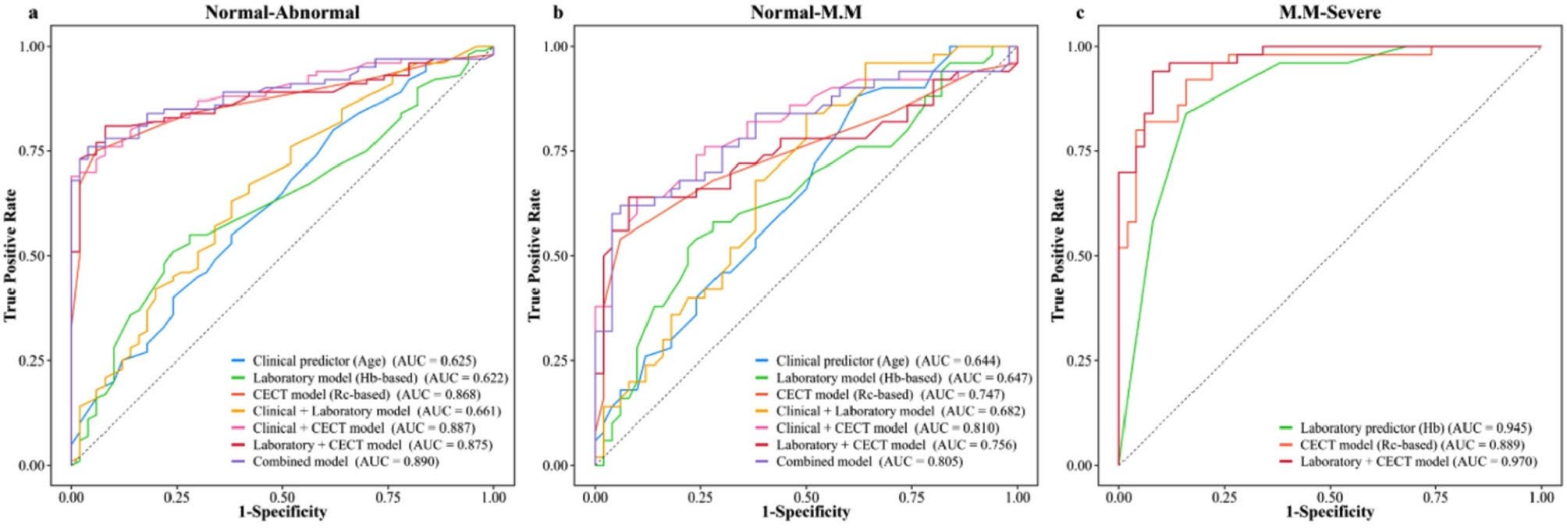
Evaluation and comparison of the diagnostic performance of different models using ROC curves for the three renal function grading prediction tasks: **(a)** Normal vs. abnormal renal function, **(b)** Normal vs. mild-to-moderate impairment, and **(c)** Mild-to-moderate vs. severe impairment. ROC = receiver operating characteristic; AUC = area under the receiver operating characteristic curve; Hb = hemoglobin; Rc = renal cortical thickness; M.M = mild-to-moderate

**Fig. 6:**
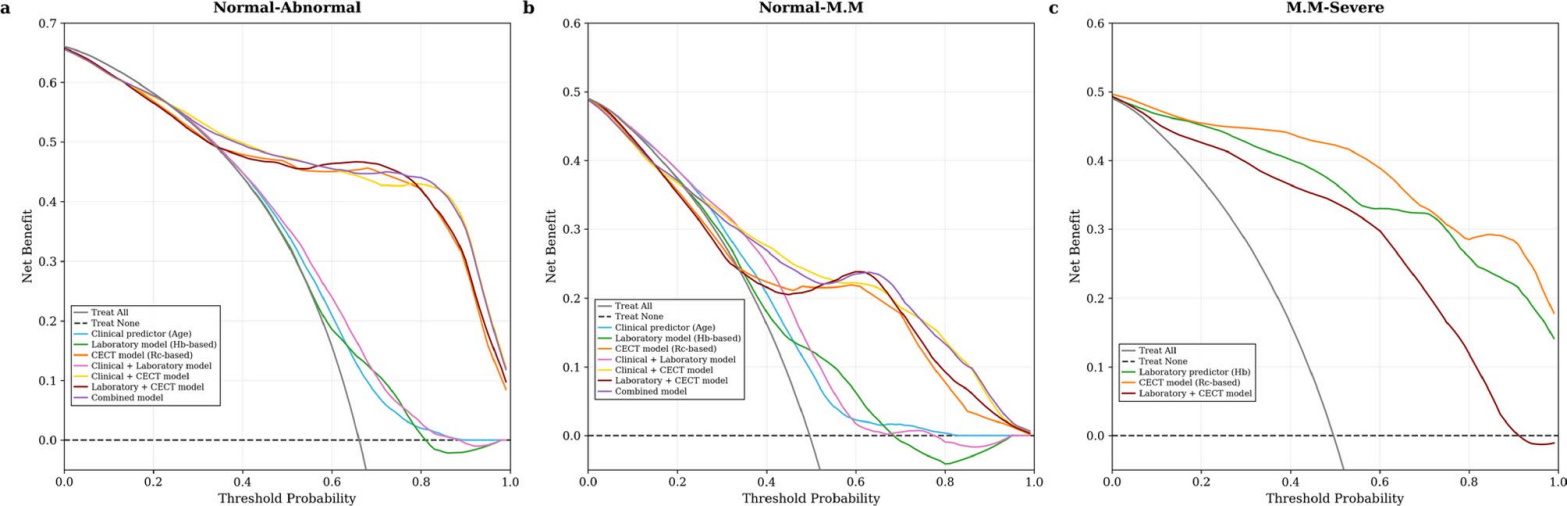
Decision curve analysis of different models for classifying kidney function. **(a)** Normal vs. abnormal renal function, **(b)** Normal vs. mild-to-moderate impairment, and **(c)** Mild-to-moderate vs. severe impairment. Hb = hemoglobin; Rc = renal cortical thickness; M.M = mild-to-moderate

Based on key predictive variables identified through univariate and multivariate analyses (including age, Hb, and Rc), we developed multiple prediction models consisting of individual variable/model, inter-model combinations, and variable-model integrations, and systematically evaluated their diagnostic performance across three renal function grading tasks. Model performance across tasks is summarized in Table 6. Across all tasks, CECT model (Rc-based) consistently demonstrated the most stable discriminative performance among imaging-derived variables. ROC and DCA further confirmed the robust performance of Rc-based models across all classification tasks (Figs. 5 and 6).

**Table 6:** Diagnostic performance of different models for three renal function grading prediction tasks

Models	Task 1: Normal-Abnormal					Models	Task 2: Normal-M.M					Models	Task 3: M.M-Severe				
	AUC	SEN	SPE	Cut-off value	*p* ^*^		AUC	SEN	SPE	Cut-off value	*p* ^*^		AUC	SEN	SPE	Cut-off value	*p* ^*^
Clinical predictor (Age)	0.625	0.800	0.380	0.625	*< 0.001*	Clinical predictor (Age)	0.644	0.880	0.380	0.431	*0.002*						
Laboratory model (Hb-based)	0.622	0.510	0.760	0.689	*< 0.001*	Laboratory model (Hb-based)	0.647	0.520	0.780	0.535	*0.015*	Laboratory predictor (Hb)	0.945	0.800	0.960	0.668	0.112
CECT model (Rc-based)	0.868	0.750	0.940	0.600	0.098	CECT model (Rc-based)	0.747	0.540	0.940	0.538	*0.046*	CECT model (Rc-based)	0.889	0.840	0.840	0.407	*0.003*
Clinical + Laboratory model	0.661	0.670	0.580	0.658	*< 0.001*	Clinical + Laboratory model	0.682	0.840	0.500	0.460	*0.016*						
Clinical + CECT model	0.887	0.690	1.000	0.837	0.654	Clinical + CECT model	**0.810**	0.640	0.900	0.604	—						
Laboratory + CECT model	0.875	0.810	0.920	0.630	0.266	Laboratory + CECT model	0.756	0.640	0.920	0.572	0.122	Laboratory + CECT model	**0.970**	0.940	0.920	0.522	—
Combined model	**0.890**	0.760	0.960	0.730	—	Combined model	0.805	0.620	0.940	0.628	0.639						

Variables were derived from the specified models. For Tasks 1 and 2, the analyses involved: Age (univariable), an Hb-based laboratory model (multivariable), and an Rc-based CECT model (multivariable). In these tasks, the column headings “Clinical”, “Laboratory”, and “CECT” correspond to Clinical predictor (Age), Laboratory model (Hb-based), and CECT model (Rc-based), respectively. For Task 3, the analyses comprised Hb (univariable) and the Rc-based CECT model (multivariable). Here, “Laboratory” refers to Laboratory predictor (Hb), and “CECT” refers to CECT model (Rc-based). The Combined model is defined as the integration of Clinical predictor (Age), Laboratory model (Hb-based), and CECT model (Rc-based). Abbreviations: Hb, hemoglobin; Rc, renal cortical thickness; M.M, mild-to-moderate; SEN, sensitivity; SPE, specificity. ^*^ DeLong’s test between the highest AUC model (presented in bold) and other models. *p* < 0.05 is indicated by italics

Pairwise comparisons of models across the three renal function classification tasks were conducted using DeLong’s test (refer to Supplementary Tables 3–5), revealing distinct patterns of diagnostic performance. In Task 1, the Combined model [Clinical predictor (Age) + Laboratory model (Hb-based) + CECT model (Rc-based)] exhibited improved performance compared with Clinical predictor (Age), Laboratory model (Hb-based) and “Clinical predictor (Age) + Laboratory model (Hb-based)” (all *p* < 0.05), with the greatest improvement observed over the Laboratory model (Hb-based) (ΔAUC = 0.268, *p* < 0.001). Notably, models incorporating CECT model (Rc-based) consistently outperformed those without (*p* < 0.001), highlighting the substantial contribution of its features to predictive performance. In Task 2, Clinical + CECT model [Clinical predictor (Age) + CECT model (Rc-based)] demonstrated optimal performance (AUC = 0.810), with AUC improvements of 0.166, 0.163, and 0.063 compared to Clinical predictor (Age), Laboratory model (Hb-based), and CECT model (Rc-based) alone, respectively (all *p* < 0.05 by DeLong’s test). Additionally, Clinical + CECT model significantly outperformed Laboratory + CECT model [Laboratory model (Hb-based) + CECT model (Rc-based)] (ΔAUC = 0.128, *p* = 0.016). These findings suggest a synergistic effect between age and Rc in identifying early renal function impairment. In Task 3, the Laboratory + CECT model [Laboratory predictor (Hb) + CECT model (Rc-based)] achieved the highest AUC, significantly outperforming CECT model (Rc-based) alone (ΔAUC = 0.081, *p* = 0.003). However, no significant difference was observed compared to Laboratory predictor (Hb) alone (ΔAUC = 0.025, *p* = 0.112). The bootstrap validation demonstrated that model performance remained stable across resampled datasets. Full validation results for all models are presented in Supplementary Table 6.

DCA further demonstrated that, in Tasks 1 and 2, the Combined model and Clinical + CECT model offered greater net benefit across a broad range of threshold probabilities (approximately 25%–100%), although they did not consistently outperform all other models. In Task 3, the Laboratory + CECT model —corresponding to the highest AUC—exhibited the greatest net benefit across the entire range of threshold probabilities.

## Discussion

This study developed and validated an integrated, noninvasive model for SRF assessment in patients with UTO by combining quantitative CECT-derived features with routine clinical and laboratory variables. Across all tasks, Rc derived from CECT consistently demonstrated stable discriminative ability. For Task 1 (normal vs. abnormal) and Task 2 (normal vs. mild-to-moderate), age was the key univariate predictor, while Hb and Rc were identified as independent predictors in laboratory-based and CECT-based multivariate models, respectively. For Task 3 (mild-to-moderate vs. severe), Hb was the strongest univariate predictor, and Rc remained the only independent imaging predictor. Notably, the models with the highest diagnostic performance across all tasks (AUC = 0.890, 0.810, and 0.970, respectively) consistently incorporated the Rc feature. Specifically, the optimal performance was achieved by the Combined model [Clinical predictor (Age) + Laboratory model (Hb-based) + CECT model (Rc-based)] in Task 1, the Clinical + CECT model [Clinical predictor (Age) + CECT model (Rc-based)] in Task 2, and the Laboratory + CECT model [Laboratory predictor (Hb) + CECT model (Rc-based)] in Task 3. These findings indicate that Rc could serve as a valuable imaging biomarker for SRF assessment, and that the integration of quantitative CECT features with clinical and laboratory parameters may offer a promising noninvasive strategy for renal function stratification.

The SRF grading thresholds were defined using age-stratified GFR reference ranges, reflecting the well-established physiological decline in renal function with aging (approximately 6.3 mL/min/1.73 m^2^ per decade) [11]. As this classification scheme inherently incorporates age into the outcome definition, the predictive role of age should be interpreted with a predictive modeling framework rather than a causal inference context [12], without implying a causal relationship. Age emerged as a significant predictor in differentiating normal from abnormal renal function (Task 1) and normal from mild-to-moderate impairment (Task 2) in univariate analysis. However, it was not significantly associated with distinguishing mild-to-moderate from severe impairment (Task 3) in univariate analysis. This pattern suggests that while aging contributes to early functional decline, severe obstructive nephropathy is predominantly driven by obstruction-related pathophysiological factors—such as etiology, duration, severity, and infection—rather than chronological age alone [1].

Renal anemia is a common complication of renal impairment, resulting from decreased erythropoietin production, toxin accumulation, and shortened erythrocyte lifespan [13]. In this study, Laboratory model (Hb-based) demonstrated independent predictive value for Tasks 1 and 2, and Laboratory predictor (Hb) emerged as the strongest univariate predictor in Task 3. However, in Tasks 1 and 2, the Laboratory model (Hb-based) alone yielded significantly lower AUCs than the Combined model. This finding highlights the importance of multimodal integration in improving diagnostic accuracy in distinguishing normal renal function from abnormal or mildly-to-moderately impaired function. In Task 3, although the Laboratory + CECT model achieved the highest AUC, no significant improvement was observed compared to the Laboratory predictor (Hb) alone (ΔAUC = 0.025, *p* = 0.112). This may be attributed to the strong independent predictive capacity of Hb for severe renal impairment, reflecting the well-established pathophysiological link between advanced chronic kidney disease and renal anemia. Nevertheless, as a marker of systemic status, Hb cannot assess asymmetric impairment between bilateral kidneys. Therefore, integrating imaging parameters that provide anatomical information into combined predictive models remains warranted, consistent with the principles of radionuclide-based GFR measurement.

Among all quantitative CECT-derived parameters, Rc showed consistent predictive performance across SRF grading tasks, particularly in differentiating mild-to-moderate from severe impairment, with an AUC of 0.889. This observation is consistent with renal physiology, as the cortex contains the majority of nephrons—including glomeruli and proximal tubules—and thus best reflects overall structural and functional integrity. Chronic obstruction leads to elevated intrapelvic pressure, cortical compression, ischemia, tubular atrophy, and fibrosis, culminating in cortical thinning [14]. In this study, mean cortical thickness declined markedly from 7 mm in the normal group to 2 mm in the severe impairment group (*p* < 0.001), confirming Rc as a sensitive biomarker of SRF deterioration.

In contrast, RPV was not an independent predictor of SRF in multivariate models, despite showing correlations in univariate analysis. This finding differs from reports in non-obstructive renal disease [15, 16] and likely reflects the unique pathophysiology of obstructive uropathy. Severe hydronephrosis can distort parenchymal boundaries and overestimate volume, while post-obstructive fibrosis introduces nonfunctional tissue into volumetric measurements [17, 18]. Additionally, compensatory hyperfiltration in the contralateral kidney may mask volume loss in the affected kidney [19]. Consequently, RPV represents a mixture of functional and nonfunctional tissue and has limited value as a surrogate for viable nephron mass in obstructed kidneys.

Rc-centered combined models demonstrated strong and consistent discriminative performance across all three SRF classification tasks (AUC range: 0.756–0.970), with optimal feature combinations varying by disease stage. The Combined model performed best in Task 1, Clinical + CECT model in Task 2, and Laboratory + CECT model in Task 3. These results indicate that optimal model composition depends not only on the individual predictor strength but also on the stage-specific pathophysiological mechanisms. In Task 2, the inclusion of age improved predictive accuracy, likely because it reflects physiological declines in renal functional reserve, enhancing detection of early impairment. In contrast, Task 3 relied more heavily on parameters reflecting systemic dysfunction (Hb) and structural injury (Rc), with the Laboratory + CECT model combination providing near-perfect classification performance.

DCA further supported the clinical utility of these combined models. In Tasks 1 and 2, the Combined model and Clinical + CECT model offered higher net benefit across a wide range of threshold probabilities (approximately 25%–100%), supporting their practical applicability. In Task 3, although the AUC difference between the Laboratory + CECT model and Laboratory predictor (Hb) was not statistically significant by the DeLong’s test, the Laboratory + CECT model demonstrated more stable and clinically meaningful net benefit. Collectively, these results emphasize the potential of Rc-based combined models for reliable SRF risk stratification and individualized clinical decision-making.

Compared with prior studies in urothelial carcinoma–related UTO, which emphasized combining renal volume with enhancement-based features to predict postoperative GFR [20], our findings demonstrate that CECT model (Rc-based) alone provides robust predictive performance across a broader etiological spectrum, with greater clinical simplicity. This highlights the practical value of CECT-derived anatomical parameters for SRF assessment. Future studies integrating cortical thickness with perfusion or radiomic features may further enhance predictive accuracy.

This study has several limitations. First, the relatively small sample size, lack of external validation, and potential selection bias limit the generalizability of the findings. Additionally, no adjustment was made for the within-subject correlation of bilateral kidneys, which may reduce statistical efficiency and introduce bias. To mitigate these concerns, bootstrap resampling (1,000 iterations) was performed for internal validation, confirming the stability of model performance across repeated sampling. Nevertheless, external validation in larger, multicenter cohorts is warranted. Second, nuclear renal dynamic imaging is subject to technical variability, particularly in cases of severe renal dysfunction. Incorporating multimodal functional imaging to establish a composite reference standard may improve robustness. Third, incomplete anthropometric data, especially missing height measurements, precluded body mass index calculation and adjustment for nutrition-related confounders, underscoring the need for more comprehensive data collection. Fourth, this study focused exclusively on morphological features; severe hydronephrosis may cause structural distortion and measurement error, and the integration of radiomic features may further enhance predictive performance. Finally, the present study establishes a predictive modeling framework rather than a directly deployable clinical tool; prospective validation and the development of a user-friendly tool (e.g., nomogram or decision-support calculators) are required before clinical implementation.

## Conclusion

This study underscores the value of renal cortical thickness (Rc) measured on contrast-enhanced CT (CECT) for split renal function (SRF) stratification in urinary tract obstruction (UTO). Rc-based models showed improved discriminative ability across different classification tasks, with age and hemoglobin (Hb) providing additional task-dependent information. The proposed approach represents a feasible, noninvasive strategy that may complement radionuclide imaging in clinical SRF evaluation.

## Supplementary Information

Below is the link to the electronic supplementary material.

Supplementary Material 1

## Data Availability

The datasets used and/or analysed during the current study are available from the corresponding author on reasonable request.

## Abbreviations

ANOVA: Analysis of variance
AUC: Area under the curve
BUN: Blood urea nitrogen
CECT: Contrast-enhanced computed tomography
CI: Confidence interval
DCA: Decision curve analysis
DICOM: Digital Imaging and Communications in Medicine
DSC: Dice similarity coefficient
eGFR: Estimated glomerular filtration rate
GFR: Glomerular filtration rate
Hb: Hemoglobin
ICC: Intraclass correlation coefficient
LLN: Lower limit of normal
MRI: Magnetic resonance imaging
OR: Odds ratio
Rc: Renal cortical thickness
R-I: Renal size index
ROC: Receiver operating characteristic
ROI: Region of interest
Rp: Renal parenchymal thickness
Rp-I: Renal parenchymal thickness index
RPV: Renal parenchymal volume
Scr: Serum creatinine
SEN: Sensitivity
SPE: Specificity
SRF: Split renal function
^99m^Tc-DTPA: ^99m^Tc-diethylenetriaminepentaacetic acid
UTO: Urinary tract obstruction
VIF: Variance inflation factor
VOI: Volume of interest
WBC: White blood cell
