# Supplementary material for: Integrating clinical, laboratory and quantitative CT features for predicting split renal function in urinary tract obstruction
Source: BMC Med Imaging. 2026 Apr 16;26:269. doi: 10.1186/s12880-026-02352-w (PMC13202847; doi:10.1186/s12880-026-02352-w)
Supplement: Supplementary file 1 — Supplementary Material 1 [file 12880_2026_2352_MOESM1_ESM.docx]

**Supplementary Information:**

1. **Supplementary Table 1.** STROBE Statement: checklist of items for the reporting of cohort studies
2. **Supplementary Table 2.** Collinearity diagnosis among the three tasks
3. **Supplementary Table 3.** DeLong's test for comparing the AUC of models in differentiating normal from abnormal renal function
4. **Supplementary Table 4.** DeLong's test for comparing the AUC of models in differentiating normal from mild-to-moderate renal function impairment
5. **Supplementary Table 5.** DeLong's test for comparing the AUC of models in differentiating mild-to-moderate from severe renal function impairment
6. **Supplementary Table 6.** Bootstrap internal validation of diagnostic performance across predictive models for hierarchical renal function grading tasks

**Supplementary Table 1.** STROBE Statement: checklist of items for the reporting of cohort studies

|  | Item No. | Recommendation | Page  No. | Relevant text from manuscript |
| --- | --- | --- | --- | --- |
| **Title and abstract** | 1 | (*a*) Indicate the study’s design with a commonly used term in the title or the abstract | 2 | A retrospective cohort of 78 patients with UTO (150 kidneys) was analyzed. |
|  |  | (*b*) Provide in the abstract an informative and balanced summary of what was done and what was found | 2-3 | Comment: The manuscript includes a structured abstract encompassing Background, Methods, Results, and Conclusion. |
| Introduction | | | |  |
| Background/rationale | 2 | Explain the scientific background and rationale for the investigation being reported | 4 | Nevertheless, most existing studies have focused on simple correlations between individual imaging parameters and renal function, without developing integrated predictive models that combine quantitative CECT features with clinical and laboratory variables. |
| Objectives | 3 | State specific objectives, including any prespecified hypotheses | 5 | Therefore, this study aimed to develop and validate predictive models for SRF grading in patients with UTO by integrating the quantitative morphological features extracted from routine CECT images with key clinical and laboratory parameters. |
| Methods | | | |  |
| Study design | 4 | Present key elements of study design early in the paper | 5 | Between January 2017 and August 2020, medical records of 111 consecutive patients with a clinical diagnosis of UTO were retrospectively reviewed. |
| Setting | 5 | Describe the setting, locations, and relevant dates, including periods of recruitment, exposure, follow-up, and data collection | 6 | Clinical and laboratory data were retrieved from the hospital’s electronic medical record system. |
| Participants | 6 | (*a*) *Cohort study*—Give the eligibility criteria, and the sources and methods of selection of participants. Describe methods of follow-up  *Case-control study*—Give the eligibility criteria, and the sources and methods of case ascertainment and control selection. Give the rationale for the choice of cases and controls  *Cross-sectional study*—Give the eligibility criteria, and the sources and methods of selection of participants | 5 | Comment: Detailed selection criteria are provided in the “Patients” subsection. Eligible adults had clinically diagnosed UTO and had undergone both contrast-enhanced CT and renal scintigraphy. Major exclusions included other renal parenchymal diseases, severe confounders of renal function, or indwelling urinary devices. |
|  |  | (*b*) *Cohort study*—For matched studies, give matching criteria and number of exposed and unexposed  *Case-control study*—For matched studies, give matching criteria and the number of controls per case | N/A | N/A |
| Variables | 7 | Clearly define all outcomes, exposures, predictors, potential confounders, and effect modifiers. Give diagnostic criteria, if applicable | 6-8 | Comment: All key variables are explicitly defined in the Methods section. The primary outcome is split renal function (SRF) grade, determined by ⁹⁹ᵐTc-DTPA scintigraphy (Gates method) and categorized using age-specific thresholds (Table 1). Predictors encompass clinical, laboratory, and CECT-derived features (e.g., age, Hb, Rc). Potential confounders were addressed in the eligibility criteria. |
| Data sources/ measurement | 8^*^ | For each variable of interest, give sources of data and details of methods of assessment (measurement). Describe comparability of assessment methods if there is more than one group | 6-9 | Comment: All variables are explicitly sourced and measured per Methods. Clinical/lab data from electronic medical record system; SRF by scintigraphy (Gates method); CECT features by blinded radiologists with reproducibility assessed (ICC/DSC). |
| Bias | 9 | Describe any efforts to address potential sources of bias | 5-9 | Comment: Major biases were mitigated by strict eligibility criteria, blinding of CECT assessors, standardized/duplicate measurements with reproducibility assessment (ICC/DSC). |
| Study size | 10 | Explain how the study size was arrived at | 5-6 | Between January 2017 and August 2020, medical records of 111 consecutive patients with a clinical diagnosis of UTO were retrospectively reviewed... After screening, 33 patients were excluded... leaving 78 eligible patients for final analysis. Six patients had a solitary kidney due to prior nephrectomy, resulting in a total of 150 kidneys available for analysis. |
| Quantitative variables | 11 | Explain how quantitative variables were handled in the analyses. If applicable, describe which groupings were chosen and why | 6-8 | Comment: Continuous variables were analyzed per their distribution. The outcome was categorized into three age-stratified groups (see Table 1). Predictors were grouped (clinical/lab/CECT) for regression modeling. |
| Statistical methods | 12 | (*a*) Describe all statistical methods, including those used to control for confounding | 9 | Comment: All statistical methods, including details on confounding control, are comprehensively described in the “Statistical analysis” subsection of the Methods. This includes group comparisons, model building via multivariate logistic regression, and assessment of multicollinearity (VIF < 5) and model performance (ROC, DCA). |
|  |  | (*b*) Describe any methods used to examine subgroups and interactions | N/A | Comment: No formal subgroup or interaction analyses were conducted. The analysis focused on developing predictive models for the entire cohort across three defined classification tasks (i.e., Task 1: normal vs. abnormal; Task 2: normal vs. mild-to-moderate; Task 3: mild-to-moderate vs. severe). |
|  |  | (*c*) Explain how missing data were addressed | N/A | Comment: Missing data were not an issue for the primary analysis. As specified in the inclusion criteria, only patients with complete clinical, laboratory, and imaging data required for the study objectives were eligible. |
|  |  | (*d*) *Cohort study*—If applicable, explain how loss to follow-up was addressed  *Case-control study*—If applicable, explain how matching of cases and controls was addressed  *Cross-sectional study*—If applicable, describe analytical methods taking account of sampling strategy | N/A | N/A |
|  |  | (*e*) Describe any sensitivity analyses | N/A | N/A |
| Results | | | | |
| Participants | 13^*^ | (a) Report numbers of individuals at each stage of study—eg numbers potentially eligible, examined for eligibility, confirmed eligible, included in the study, completing follow-up, and analysed | 5-6 | Comment: Of 111 consecutive patients reviewed, 33 were excluded, yielding 78 patients (150 kidneys) for analysis. (See Methods “Patients” and Fig. 1). |
|  |  | (b) Give reasons for non-participation at each stage | 5-6 | After screening, 33 patients were excluded (32 with coexisting renal parenchymal lesions and 1 with a ureteral stent), leaving 78 eligible patients for final analysis. |
|  |  | (c) Consider use of a flow diagram | 6 | The patient selection process is summarized in Fig. 1. |
| Descriptive data | 14^*^ | (a) Give characteristics of study participants (eg demographic, clinical, social) and information on exposures and potential confounders | 10 | A total of 150 kidneys from 78 patients with UTO (39 males and 39 females; median age, 59.0 years) were included... No significant intergroup differences were observed in sex, body weight, obstruction etiology, or laterality (all *p* > 0.05). In contrast, age and laboratory parameters—including Hb, Scr, BUN, and eGFR—differed significantly among the three renal function groups (all *p* < 0.05). Detailed clinical and laboratory characteristics are summarized in Table 2. |
|  |  | (b) Indicate number of participants with missing data for each variable of interest | N/A | Comment: No missing data were present for all study variables. |
|  |  | (c) *Cohort study*—Summarise follow-up time (eg, average and total amount) | N/A | N/A |
| Outcome data | 15^*^ | *Cohort study*—Report numbers of outcome events or summary measures over time | N/A | Comment: This is a cross-sectional diagnostic study. The outcome (SRF grade) and predictors were assessed at a single time point; thus, longitudinal outcome measures are not applicable. |
|  |  | *Case-control study—*Report numbers in each exposure category, or summary measures of exposure | N/A | N/A |
|  |  | *Cross-sectional study—*Report numbers of outcome events or summary measures | N/A | N/A |
| Main results | 16 | (*a*) Give unadjusted estimates and, if applicable, confounder-adjusted estimates and their precision (eg, 95% confidence interval). Make clear which confounders were adjusted for and why they were included | 10-11 | Comment: Both unadjusted and adjusted odds ratios (OR) with 95% confidence intervals are presented in Tables 3-5. The multivariable models included variables that were significant in univariate analysis and showed low collinearity (VIF < 5). |
|  |  | (*b*) Report category boundaries when continuous variables were categorized | 6-7 | According to the age-specific lower limit of normal (LLN) for total GFR provided in the GE operator manual, kidneys were initially categorized as having either normal or abnormal function. The abnormal group was further stratified into mild, moderate, and severe impairment based on the following criteria (refer to Table 1): Normal, total GFR ≥ LLN; Mild impairment, < LLN but ≥ 2/3 LLN; Moderate impairment, < 2/3 LLN but ≥ 1/3 LLN; and Severe impairment, < 1/3 LLN. The reference value for SRF was defined as one-half of the age-specific total GFR reference value. This grading scheme reflects physiological age-related variation in renal function and provides a standardized framework for SRF stratification. |
|  |  | (*c*) If relevant, consider translating estimates of relative risk into absolute risk for a meaningful time period | N/A | N/A |
| Other analyses | 17 | Report other analyses done—eg analyses of subgroups and interactions, and sensitivity analyses | N/A | Comment: No subgroup, interaction, or sensitivity analyses were conducted. |
| Discussion | | | | |
| Key results | 18 | Summarise key results with reference to study objectives | 13 | These findings indicate that Rc could serve as a valuable imaging biomarker for SRF assessment, and that the integration of quantitative CECT features with clinical and laboratory parameters may offer a promising noninvasive strategy for renal function stratification. |
| Limitations | 19 | Discuss limitations of the study, taking into account sources of potential bias or imprecision. Discuss both direction and magnitude of any potential bias | 15 | Comment: Study limitations, including potential biases and their implications (e.g., sample size, selection bias, reference standard variability, unmeasured confounding, measurement error, lack of external validation), are discussed in detail in the final paragraph of the Discussion. |
| Interpretation | 20 | Give a cautious overall interpretation of results considering objectives, limitations, multiplicity of analyses, results from similar studies, and other relevant evidence | 3, 15-16 | Comment: The cautious overall interpretation is stated in the Abstract Conclusion (“This integrated, noninvasive strategy offers a practical adjunct to radionuclide imaging for individualized SRF assessment”) and reiterated in the Discussion, acknowledging limitations and the need for future validation. |
| Generalisability | 21 | Discuss the generalisability (external validity) of the study results | 15 | Finally, the absence of external validation limits generalizability and clinical applicability, highlighting the need for confirmation in independent, multicenter cohorts. |
| Other information | |  | | |
| Funding | 22 | Give the source of funding and the role of the funders for the present study and, if applicable, for the original study on which the present article is based | 17 | This work was supported by the National Natural Science Foundations of China (No. 82371908, 62262029), the Guangdong Natural Science Foundation (No. 2024A1515012177), the Jiangxi Provincial Natural Science Foundation Project (No. 20242BAB25546), 2025 Research Project of Guangdong Health Information Network Association (No. MS-202509-0047), and Medical Science and Technology Research Project of Guangdong Province (No. A2024637). |

N/A: not applicable; STROBE: Strengthening the Reporting of Observational Studies in Epidemiology.

^*^Give information separately for cases and controls in case-control studies and, if applicable, for exposed and unexposed groups in cohort and cross-sectional studies.

| **Supplementary Table.2** Collinearity diagnosis among the three tasks | | | | | | | | |
| --- | --- | --- | --- | --- | --- | --- | --- | --- |
| **Variables** | **Normal-Abnormal** | |  | **Normal-M.M** | |  | **M.M-Severe** | |
|  | **VIF** | **Tolerance** |  | **VIF** | **Tolerance** |  | **VIF** | **Tolerance** |
| **Clinical factors** |  |  |  |  |  |  |  |  |
| Age | 1.919 | 0.521 |  | 1.865 | 0.536 |  | 1.265 | 0.791 |
| **Laboratory parameters** |  |  |  |  |  |  |  |  |
| Hb | 1.497 | 0.668 |  | 1.586 | 0.630 |  | 2.463 | 0.406 |
| Scr | 7.190 | 0.139 |  | 6.645 | 0.150 |  | 3.440 | 0.291 |
| BUN | 3.502 | 0.286 |  | 3.080 | 0.325 |  | 3.183 | 0.314 |
| eGFR | 4.207 | 0.238 |  | 4.333 | 0.231 |  | 2.313 | 0.432 |
| **CECT features** |  |  |  |  |  |  |  |  |
| RPV | 2.798 | 0.357 |  | 2.477 | 0.404 |  | 2.373 | 0.421 |
| Rc | 5.599 | 0.179 |  | 3.316 | 0.302 |  | 4.954 | 0.202 |
| Rp | 877.662 | 0.001 |  | 448.175 | 0.002 |  | 763.875 | 0.001 |
| Rp-I | 864.599 | 0.001 |  | 445.000 | 0.002 |  | 754.435 | 0.001 |
| Multicollinearity was diagnosed using the variance inflation factor (VIF), with a threshold of 5 (Tolerance < 0.1). M.M, mild-to-moderate; RPV, renal parenchymal volume; Rc, renal cortical thickness; Rp, renal parenchymal thickness; Rp-I, renal parenchymal thickness index; Hb, hemoglobin; Scr, serum creatinine; BUN, blood urea nitrogen; eGFR, estimated glomerular filtration rate. | | | | | | | | |
|  |  |  |  |  |  |  |  |  |
|  |  |  |  |  |  |  |  |  |
|  |  |  |  |  |  |  |  |  |

| **Supplementary Table.3** DeLong's test for comparing the AUC of models in differentiating normal from abnormal renal function | | | | | | |
| --- | --- | --- | --- | --- | --- | --- |
| **Model 1** | **Model 2** | **ΔAUC** | **SE** | **95% CI** | ***z*** | ***p*** |
| Clinical + Laboratory model | Clinical + CECT model | -0.226 | 0.048 | 0.133–0.320 | 4.754 | **< 0.001** |
| Clinical + Laboratory model | Laboratory + CECT model | -0.214 | 0.053 | 0.111–0.318 | 4.051 | **< 0.001** |
| Clinical + Laboratory model | Combined model | -0.229 | 0.047 | 0.137–0.321 | 4.891 | **< 0.001** |
| Clinical + Laboratory model | Laboratory model (Hb-based) | 0.039 | 0.039 | -0.038–0.115 | 0.985 | 0.325 |
| Clinical + Laboratory model | CECT model (Rc-based) | 0.207 | 0.055 | 0.099–0.315 | 3.766 | **< 0.001** |
| Clinical + Laboratory model | Clinical predictor (Age) | 0.036 | 0.033 | -0.029–0.101 | 1.085 | 0.278 |
| Clinical + CECT model | Laboratory + CECT model | 0.012 | 0.015 | -0.017–0.042 | 0.817 | 0.414 |
| Clinical + CECT model | Combined model | -0.003 | 0.005 | -0.007–0.011 | 0.449 | 0.654 |
| Clinical + CECT model | Laboratory model (Hb-based) | 0.265 | 0.052 | 0.164–0.367 | 5.145 | **< 0.001** |
| Clinical + CECT model | CECT model (Rc-based) | 0.019 | 0.014 | -0.007–0.047 | 1.422 | 0.155 |
| Clinical + CECT model | Clinical predictor (Age) | 0.262 | 0.048 | 0.168–0.357 | 5.445 | **< 0.001** |
| Laboratory + CECT model | Combined model | -0.015 | 0.013 | -0.011–0.040 | 1.113 | 0.266 |
| Laboratory + CECT model | Laboratory model (Hb-based) | 0.253 | 0.049 | 0.157–0.349 | 5.162 | **< 0.001** |
| Laboratory + CECT model | CECT model (Rc-based) | 0.007 | 0.006 | -0.004–0.019 | 1.232 | 0.218 |
| Laboratory + CECT model | Clinical predictor (Age) | 0.250 | 0.058 | 0.138–0.363 | 4.356 | **< 0.001** |
| Combined model | Laboratory model (Hb-based) | 0.268 | 0.049 | 0.171–0.364 | 5.448 | **< 0.001** |
| Combined model | CECT model (Rc-based) | 0.022 | 0.013 | -0.004–0.048 | 1.656 | 0.098 |
| Combined model | Clinical predictor (Age) | 0.265 | 0.049 | 0.168–0.361 | 5.368 | **< 0.001** |
| Laboratory model (Hb-based) | CECT model (Rc-based) | -0.246 | 0.052 | 0.143–0.348 | 4.698 | **< 0.001** |
| Laboratory model (Hb-based) | Clinical predictor (Age) | -0.003 | 0.064 | -0.123–0.129 | 0.042 | 0.967 |
| CECT model (Rc-based) | Clinical predictor (Age) | 0.243 | 0.058 | 0.130–0.356 | 4.209 | **< 0.001** |
| The variates included for Task 1 (differentiating normal from abnormal renal function) were: Age (univariable), an Hb-based laboratory model (multivariable), and an Rc-based CECT model (multivariable). In this task, the column headings "Clinical," "Laboratory," and "CECT" correspond to Clinical predictor (Age), Laboratory model (Hb-based), and CECT model (Rc-based), respectively. The Combined model is defined as the integration of Clinical predictor (Age), Laboratory model (Hb-based), and CECT model (Rc-based). ΔAUC was calculated as the AUC of model 1 minus the AUC of model 2. Abbreviations: Hb, hemoglobin; Rc, renal cortical thickness; SE, standard error; *p* < 0.05 is indicated by bold type. | | | | | | |

| **Supplementary Table.4** DeLong's test for comparing the AUC of models in differentiating normal from mild-to-moderate renal function impairment | | | | | | |
| --- | --- | --- | --- | --- | --- | --- |
| **Model 1** | **Model 2** | **ΔAUC** | **SE** | **95% CI** | ***z*** | ***p*** |
| Laboratory model (Hb-based) | Clinical predictor (Age) | 0.003 | 0.078 | -0.150–0.157 | 0.041 | 0.967 |
| CECT model (Rc-based) | Clinical predictor (Age) | 0.103 | 0.075 | -0.044–0.251 | 1.370 | 0.171 |
| Clinical + Laboratory model | Clinical predictor (Age) | 0.038 | 0.038 | -0.037–0.112 | 0.994 | 0.320 |
| Clinical + CECT model | Clinical predictor (Age) | 0.166 | 0.053 | 0.063–0.269 | 3.145 | **0.002** |
| Laboratory + CECT model | Clinical predictor (Age) | 0.112 | 0.076 | -0.037–0.261 | 1.469 | 0.142 |
| Combined model | Clinical predictor (Age) | 0.161 | 0.055 | 0.054–0.268 | 2.945 | **0.003** |
| CECT model (Rc-based) | Laboratory model (Hb-based) | 0.100 | 0.064 | -0.026–0.226 | 1.556 | 0.120 |
| Clinical + Laboratory model | Laboratory model (Hb-based) | 0.035 | 0.050 | -0.064–0.133 | 0.685 | 0.494 |
| Clinical + CECT model | Laboratory model (Hb-based) | 0.163 | 0.067 | 0.032–0.293 | 2.443 | **0.015** |
| Laboratory + CECT model | Laboratory model (Hb-based) | 0.109 | 0.056 | -0.002–0.219 | 1.924 | 0.054 |
| Combined model | Laboratory model (Hb-based) | 0.158 | 0.060 | 0.040–0.275 | 2.635 | **0.008** |
| Clinical + Laboratory model | CECT model (Rc-based) | 0.058 | 0.070 | -0.071–0.202 | 0.944 | 0.345 |
| Clinical + CECT model | CECT model (Rc-based) | 0.063 | 0.032 | 0.001–0.125 | 1.995 | **0.046** |
| Laboratory + CECT model | CECT model (Rc-based) | 0.009 | 0.014 | -0.019–0.035 | 0.611 | 0.541 |
| Combined model | CECT model (Rc-based) | 0.058 | 0.030 | -0.001–0.116 | 1.939 | 0.053 |
| Clinical + CECT model | Clinical + Laboratory model | 0.128 | 0.053 | 0.024–0.233 | 2.416 | **0.016** |
| Laboratory + CECT model | Clinical + Laboratory model | 0.074 | 0.066 | -0.055–0.203 | 1.126 | 0.260 |
| Combined model | Clinical + Laboratory model | 0.123 | 0.049 | 0.026–0.220 | 2.494 | **0.013** |
| Laboratory + CECT model | Clinical + CECT model | -0.054 | 0.035 | -0.015–0.123 | 1.546 | 0.122 |
| Combined model | Clinical + CECT model | -0.005 | 0.011 | -0.017–0.027 | 0.469 | 0.639 |
| Combined model | Laboratory + CECT model | 0.049 | 0.031 | -0.011–0.109 | 1.615 | 0.106 |
| The variates included for Task 2 (differentiating normal from mild-to-moderate renal function impairment) were: Age (univariable), an Hb-based laboratory model (multivariable), and an Rc-based CECT model (multivariable). In this task, the column headings "Clinical," "Laboratory," and "CECT" correspond to Clinical predictor (Age), Laboratory model (Hb-based), and CECT model (Rc-based), respectively. The Combined model is defined as the integration of Clinical predictor (Age), Laboratory model (Hb-based), and CECT model (Rc-based). ΔAUC was calculated as the AUC of model 1 minus the AUC of model 2. Abbreviations: Hb, hemoglobin; Rc, renal cortical thickness; SE, standard error; *p* < 0.05 is indicated by bold type. | | | | | | |

| **Supplementary Table.5** DeLong's test for comparing the AUC of models in differentiating mild-to-moderate from severe renal function impairment | | | | | | |
| --- | --- | --- | --- | --- | --- | --- |
| **Model 1** | **Model 2** | **ΔAUC** | **SE** | **95% CI** | ***z*** | ***p*** |
| Laboratory + CECT model | CECT model (Rc-based) | 0.081 | 0.028 | 0.027–0.135 | 2.925 | **0.003** |
| Laboratory + CECT model | Laboratory predictor (Hb) | 0.025 | 0.016 | -0.006–0.056 | 1.590 | 0.112 |
| CECT model (Rc-based) | Laboratory predictor (Hb) | -0.056 | 0.039 | -0.022–0.132 | 1.412 | 0.158 |
| The variates included for Task 3 (differentiating mild-to-moderate from severe renal function impairment) were: Hb (univariable) and the Rc-based CECT model (multivariable). Here, "Laboratory" refers to Laboratory predictor (Hb), and "CECT" refers to CECT model (Rc-based). ΔAUC was calculated as the AUC of model 1 minus the AUC of model 2. Abbreviations: Hb, hemoglobin; Rc, renal cortical thickness; SE, standard error; *p* < 0.05 is indicated by bold type. | | | | | | |

| **Supplementary Table 6.** Bootstrap internal validation of diagnostic performance across predictive models for hierarchical renal function grading tasks | | | | | | | | | | | | | | |
| --- | --- | --- | --- | --- | --- | --- | --- | --- | --- | --- | --- | --- | --- | --- |
| **Models** | **Task 1：Normal-Abnormal** | | | | **Models** | **Task 2：Normal-M.M** | | | | **Models** | **Task 3：M.M-Severe** | | | |
|  | **Apparent**  **AUC** | **Bootstrap AUC** | **SE** | **95%CI** |  | **Apparent**  **AUC** | **Bootstrap AUC** | **SE** | **95%CI** |  | **Apparent**  **AUC** | **Bootstrap AUC** | **SE** | **95%CI** |
| Clinical predictor (Age) | 0.625 | 0.623 | 0.051 | 0.518-0.719 | Clinical predictor (Age) | 0.644 | 0.646 | 0.0538 | 0.536–0.750 |  |  |  |  |  |
| Laboratory model (Hb-based) | 0.622 | 0.620 | 0.048 | 0.520-0.711 | Laboratory model (Hb-based) | 0.647 | 0.648 | 0.0556 | 0.544–0.749 | Laboratory predictor (Hb) | 0.945 | 0.944 | 0.0222 | 0.895–0.983 |
| CECT model (Rc-based) | 0.868 | 0.866 | 0.029 | 0.804–0.917 | CECT model (Rc-based) | 0.747 | 0.746 | 0.0508 | 0.647–0.835 | CECT model (Rc-based) | 0.889 | 0.889 | 0.0334 | 0.822–0.950 |
| Clinical + Laboratory | 0.661 | 0.663 | 0.048 | 0.566–0.754 | Clinical + Laboratory | 0.682 | 0.683 | 0.0545 | 0.574–0.788 |  |  |  |  |  |
| Clinical + CECT | 0.887 | 0.886 | 0.027 | 0.827–0.934 | Clinical + CECT | **0.810** | **0.810** | 0.0438 | 0.717–0.892 |  |  |  |  |  |
| Laboratory + CECT | 0.875 | 0.875 | 0.028 | 0.820–0.926 | Laboratory + CECT | 0.756 | 0.755 | 0.0516 | 0.646–0.852 | Laboratory + CECT | **0.970** | **0.971** | 0.0139 | 0.939–0.993 |
| Combined model | **0.890** | **0.893** | 0.027 | 0.837-0.939 | Combined model | 0.805 | 0.803 | 0.0446 | 0.710–0.888 |  |  |  |  |  |
| Variates were derived from the specified models. For Tasks 1 and 2, the analyses involved: Age (univariable), an Hb-based laboratory model (multivariable), and an Rc-based CECT model (multivariable). In these tasks, the column headings "Clinical", "Laboratory", and "CECT" correspond to Clinical predictor (Age), Laboratory model (Hb-based), and CECT model (Rc-based), respectively. For Task 3, the analyses comprised Hb (univariable) and the Rc-based CECT model (multivariable). Here, "Laboratory" refers to Laboratory predictor (Hb), and "CECT" refers to CECT model (Rc-based). The Combined model is defined as the integration of Clinical predictor (Age), Laboratory model (Hb-based), and CECT model (Rc-based). Apparent AUC was estimated from the original sample; bootstrap AUC denotes the bias-corrected estimate from 1,000 resamples, with SE and 95% CI estimated from the bootstrap distribution. Bold values indicate the highest AUC within each task. Abbreviations: Hb, hemoglobin; Rc, renal cortical thickness; M.M, mild-to-moderate; SE, standard error. | | | | | | | | | | | | | | |
